# Efficacy of a Mouthwash Containing Resveratrol in Reducing Halitosis-related P. gingivalis: A Randomized Triple-blind Trial

**DOI:** 10.3290/j.ohpd.c_2373

**Published:** 2025-12-02

**Authors:** Noor A. Abed Taher, Athraa Ali Mahmood, Hashim Mueen Hussein

**Affiliations:** a Noor A. Abed Taher MSc Student, Department of Oral Surgery and Periodontics, College of Dentistry, Mustansiriyah University, Baghdad, Iraq. Study concept, resources, materials, data collection and/or processing, literature search, wrote the manuscript, funding.; b Athraa Ali Mahmood Professor in Periodontics, Department of Oral Surgery and Periodontics, College of Dentistry, Mustansiriyah University, Baghdad, Iraq. Study concept and resources, supervision, resources, materials, data collection and/or processing, analysis and/or Interpretation, literature search, wrote and critically reviewed the manuscript.; c Hashim Mueen Hussein Lecturer, Department of Conservative Dentistry, College of Dentistry, Mustansiriyah University, Baghdad, Iraq. Analysis and/or interpretation, wrote and critically reviewed the manuscript.

**Keywords:** chlorhexidine, halitosis, mouthwash, Porphyromonas gingivalis, resveratrol.

## Abstract

**Purpose:**

To assess the efficacy of an anti-oxidant and anti-inflammatory RSV-mouthwash in reducing halitosis-related P. gingivalis and clinical periodontal parameters (plaque index [PI], gingival index [GI], and bleeding on probing [BOP]) in undergraduate dental students with plaque-induced gingivitis.

**Materials and Methods:**

This research was conducted as a randomized, triple-blind clinical trial involving 54 participants who had halitosis associated with plaque-induced gingivitis. Resveratrol (RSV) mouthwash was used by the test group, and 0.2% CHX digluconate mouthwash was used as the positive control. The mouthwash used in the negative control group was distilled water (DW). Clinical parameters, including PI, GI, and BOP, were examined at the baseline appointment and again after one week of using mouthwashes alongside routine oral hygiene measures. Porphyromonas gingivalis was chosen as the target microbe due to its known status as a major pathogen linked to periodontal disease and bad breath. The presence of P. gingivalis was evaluated and compared before and after treatment by real-time PCR. During the last appointment, the participants responded to an mouthwash-assessment questionnaire based on a visual analog scale (VAS). Data description, analysis, and presentation were performed using the SPSS, with the significance level set at p < 0.05. This trial was registered at clinicaltrials.gov (NCT06882564).

**Results:**

RSV and CHX statistically significantly reduced halitosis scores, PI, BOP, GI, and the level of P. gingivalis in plaque samples. According to participants’ answers to the mouthwash survey, there were no statistically significant differences between RSV and CHX.

**Conclusion:**

RSV mouthwash has a statistically significant effect on the treatment of P. gingivalis-related halitosis when used as an adjunct to routine oral care. RSV caused a statistically significant decrease in clinical periodontal parameters, including PI, BOP, GI, and halitosis scores, with a statistically significant reduction in the level of P. gingivalis. Thus, RSV shows promising short-term efficacy and warrants further longer-term and larger-scale studies.

Bad breath or halitosis is the third most frequently reported oral health concern in dental practice and affects up to 50% of the population.^30^ Halitosis arises mainly from the overgrowth of certain bacteria (gram-negative), such as Fusobacterium nucleatum (F. nucleatum) and Porphyromonas gingivalis (P. gingivalis), which are known to cause biofilm-associated infections – such as dental biofilm, periodontal disease and its associated systemic effects,^31,35^ – as well as producing volatile sulfur compounds (VSCs), including such malodorous gases as hydrogen sulfide, methyl mercaptan, and dimethylamine, leading to bad breath.^20^ P. gingivalis plays a significant role in dental biofilm formation.^29^ Its gingival protease causes bad breath by breaking down amino acids to create VSCs, contributing to the pathophysiology of periodontitis. Additionally, gingivitis caused by lipopolysaccharide (LPS) components of gram-negative bacteria aggravates halitosis.^5,29^ Management of halitosis and other periodontal conditions still relies on thoroughly analyzing microbial etiology.^3,4,5,6,7,8,9,10,11,12,13,14,15,16,17,18^ Currently, one of the most effective approaches for the treatment of halitosis is the elimination of harmful bacteria that generate VSCs in the oral cavity.^9^

Along with mechanical therapy, a variety of chemotherapeutic treatments have been suggested to reduce halitosis.^34^ Chlorhexidine (CHX) is a major anti-bacterial product that can inhibit a wide spectrum of pathogenic microorganisms, support dental biofilm formation, and reduce malodors. It works by penetrating the bacterial cell membrane, resulting in cell leakage and metabolic disturbances, which stop cell growth.^3,15^ However, this treatment has certain adverse effects, including taste disturbance and reversible tooth staining.^26^

Resveratrol (RSV) is a phytoalexin produced by various plants and is abundant in peanuts and red grape skins. RSV is a nutraceutical that has recently attracted considerable interest, owing to its promising pharmacological properties.^13^ It is present in several plants, including grapes, berries, and peanuts, and can defend plants against microbial and fungal infection.^36^ RSV exhibits anti-biofilm and anti-bacterial characteristics and primarily targets markers associated with inflammation and adhesion.^14^ It has anti-inflammatory, anti-oxidant, and possibly longevity effects; its anti-bacterial properties, keratinocyte barrier protection, and ability to reduce monocyte inflammatory response make it a unique treatment agent for periodontal disease.^22^

Despite being the gold standard for chemical plaque treatment and halitosis management, CHX has a number of negative effects that limit its long-term use, including mucosal irritation, tooth discoloration, and altered taste perception. On the other hand, RSV, a naturally occurring polyphenol with demonstrated antibacterial and antioxidant qualities, is a viable substitute. Its clinical importance as an alternative to CHX in the long-term therapy of halitosis is highlighted by its excellent safety profile and potential for improved patient compliance. RSV prevents the formation of P. gingivalis biofilms and reduces the expression of genes responsible for virulence factors, including fimbriae and protease enzymes.^13,37^

Previous studies have investigated RSV mainly in the form of toothpaste, dietary supplements, or as an adjunct with other agents for managing periodontal diseases or halitosis. To the best of our knowledge, the present trial is the first to evaluate resveratrol mouthwash alone as a therapeutic option for halitosis. This study aimed to assess the efficacy of an RSV-mouthwash alongside routine oral hygiene measures in halitosis treatment, in contrast to a negative control (placebo) and a positive control (CHX), and to assess the shift in plaque samples of P. gingivalis. The null hypothesis was that the RSV mouthwash does not affect halitosis.

## MATERIALS AND METHODS

### Study Design

This parallel, randomized, triple-blind clinical study was carried out with randomly selected 4^th^- and 5^th^-year undergraduate dental students at the Department of Periodontics, Mustansiriyah University’s College of Dentistry. Ethical approval was obtained in accordance with the Declaration of Helsinki.^33^

This trial was registered at clinicaltrials.gov (NCT06882564), with a link https://clinicaltrials.gov/study/NCT06882564.

### Sample Size, Inclusion and Exclusion Criteria

The sample size was calculated using G*Power (version 3.1), Cohen’s d = (M2 – M1) ⁄ SD pooled, based on the data obtained from a previous similar study,^19^ which showed that CHX 0.2% mouthwash reduced the organoleptic score (2.2 ± 0.45) more than did the placebo (3.2 ± 0.89). The difference in the effect size between the CHX and placebo was 1.4. at a probability power (β) of 80% and a statistical significance level of α = 0.05.

A sample size of 18 participants (14 calculated and 4 to compensate for dropouts) was sufficient to reject the null hypothesis. Since RSV is a novel treatment for halitosis, a formal power estimate for this group could not be completed.

The inclusion criteria were: male and female undergraduate dental students with halitosis due to gingivitis and who had not performed chemical plaque control measures for at least one week. The age of the students ranged from 20 to 23 years, who had had no periodontal treatment for at least one month, no systemic disease, and no tobacco smoking. The patients needed to have at least 20 teeth.

The exclusion criteria were: smokers and alcoholics, as well as individuals with periodontitis, orthodontic appliances, open carious lesions, pericoronitis, dry socket, or fistula, systemic diseases, and pregnancy. Furthermore, taking medication, garlic, onion, or oral hygiene products in the last 24 h led to exclusion, as did sinusitis, tonsillitis, and upper respiratory tract infections.

### Hypothesis and Outcomes

The null hypothesis stated that RSV mouthwash is ineffective in reducing the level of P. gingivalis and halitosis. The alternative hypothesis was that RSV mouthwash is effective in reducing the level of P. gingivalis and halitosis.

The primary outcomes were reducing halitosis scores measured by the organoleptic method and the halimeter, and reducing the level of P. gingivalis measured by real-time PCR. Secondary outcomes consisted of improvement of clinical periodontal parameters (PI, GI, BOP), and results of the intervention using a VAS score-based questionnaire.

### Randomization, Blinding, and Study Groups

**Fig 1:**
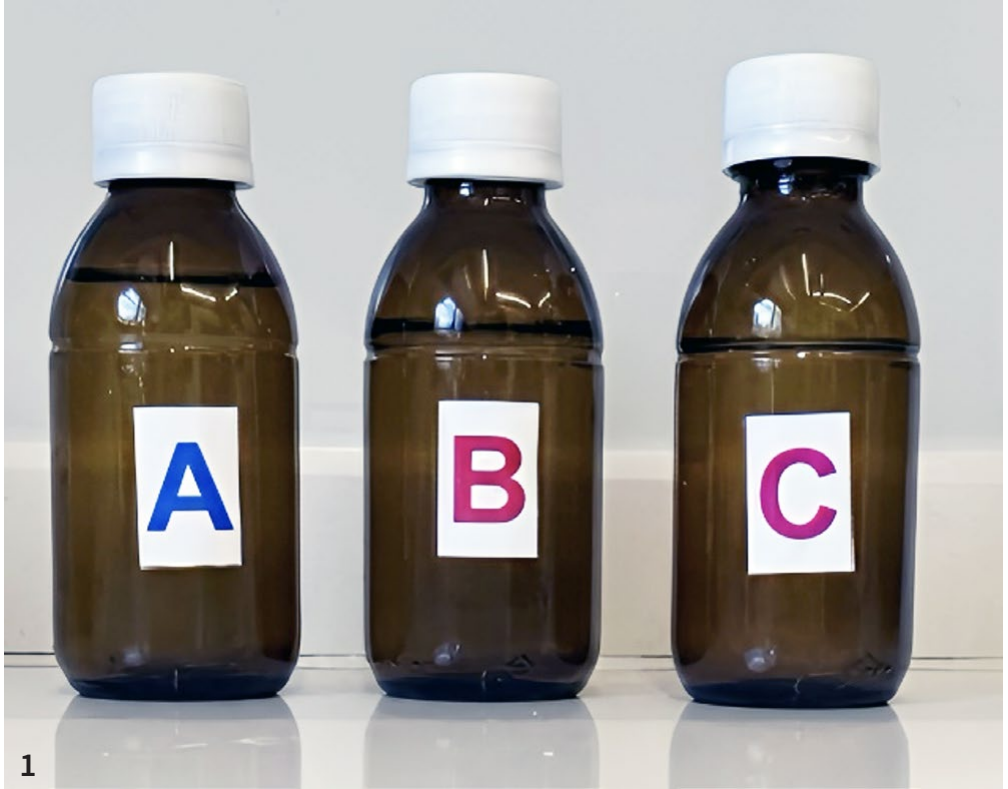
Blinding the mouthwash by letter coding.

Block randomization was used to establish the intervention sequence, and each student had an equal probability of being randomly assigned to it. DW as the negative control, 0.2% CHX as the positive control, and RSV as the test. A dentist unrelated to this study assigned the following codes to the mouthwashes – A, B, and C – and kept them in opaque bottles (Fig 1).

Oroxil mouthwash, which contains nanotechnology-enhanced RSV combined with an essential oil from wild thyme (Logidex; Turin, Italy), was used by the test group. A mouthwash containing 0.2% CHX digluconate was used as the positive control (Kin; Castlebar, Ireland), and the mouthwash used in the negative control group was distilled water (DW).

All the participants were instructed to rinse twice daily (every 12 h) with 15 ml of the assigned mouthwash (undiluted) for 30 s, after 30 min of toothbrushing. They were also provided with measuring cups with a 15-ml mark in order to use the correct volume of mouthwash. They were instructed to refrain from eating and drinking for 30 min after rinsing with no change to their routine home care (brushing and interdental cleaning aids).

### Clinical Procedure

During the initial appointment (baseline), a comprehensive medical and dental history was obtained from each student. The subjects were assessed for oral halitosis between 11 A.M. and 1 P.M. using the organoleptic tongue and floss methods on a six-point scale. Organoleptic measurements were taken by 3 tests. The tongue odor test was used to assess halitosis originating from the dorsum (top surface) of the tongue.

**Fig 2:**
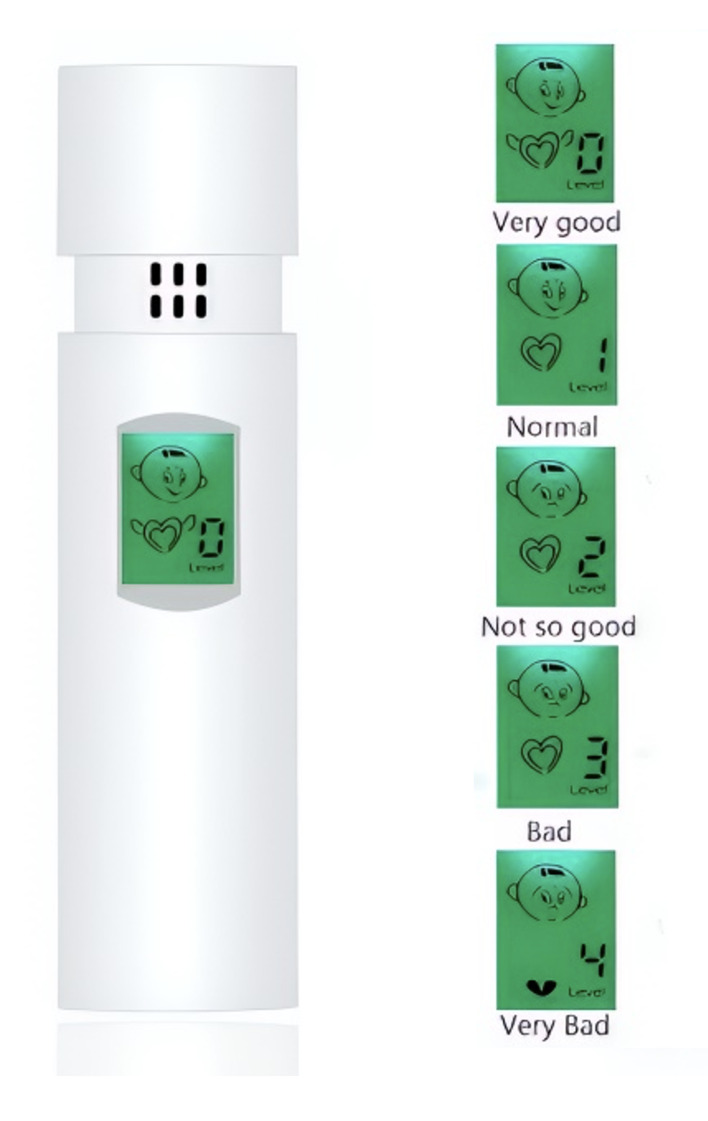
Portable halimeter.

The tongue’s posterior dorsum was scraped with a sterile cotton roll, and the odor was measured 5 s later while the cotton roll was held five centimeters from the nose.^10^ A test for the smell of dental floss was used to identify halitosis originating from the spaces between the teeth. A flossing pick was inserted into the interdental space of each tooth and removed immeditately. The floss odor was assessed by holding the pick approximately 3 cm from the nose.^24^ A portable sulfide monitor (portable halimeter) was used to confirm the results of the organoleptic method (Fig 2). The participant was instructed to blow air towards the blowing port for 3-5 s from a distance of approximately 3 cm in front of the blowing port. The results were recorded using five levels, represented by the digits 0, 1, 2, 3, 4.

The same clinical procedures were repeated at the second visit after 7 days.

### Clinical Assessment

One examiner evaluated PI, BOP, and GI using the University of North Carolina-15 (UNC-15) probes. Plaque index was taken using the O’Leary PI.^23^ At the 1st appointment, all accessible tooth surfaces were coated with a disclosing solution (Bioclear matrix; Tacoma, WA, USA).^23^ After the patient had rinsed, the examiner looked for soft accumulations in each stained area using an explorer or a probe tip. Following the examination and scoring of each tooth, the percentage of PI was determined by dividing the number of plaque-covered tooth surfaces by the total number of examined tooth surfaces and multiplying the result by 100%. BOP was assessed by gently inserting a UNC-15 probe into the gingival sulcus at six sites around each tooth, removing the probe coronally, and waiting for 30 s to check for the presence or absence of bleeding (no bleeding 0, bleeding present =1).^21^ GI was measured using the Löe and Silness index,^16^ and each of the four gingival areas of the tooth was scored from 0 to 3 as follows: 0 for normal gingiva, 1 for mild inflammation, and 3 for severe inflammation.

The same clinical procedures were repeated at the second visit after 7 days.

### Sample Collection

Supragingival plaque samples were collected from four gingival sulci with a sterile mini-curette from the buccal surfaces of the first or second molar in both the maxilla and mandible. The curette was inserted without exerting any pressure on the tooth surface, the subgingival sample was collected using a single vertical stroke and then immediately placed into an Eppendorf tube containing TE buffer (0.5 ml).

At the 2nd appointment (after 7 days), the subjects were instructed to use the mouthwash after breakfast and not to eat or drink 2 h before the assessment (11 A.M. and 1 P.M.). Halitosis was then assessed using the organoleptic tongue and floss methods, in addition to a halimeter, as described above. Clinical assessement as described above was performed again.

### Calibration

Inter- and intra-examiner calibration was performed for the clinical periodontal parameters PI, BOP, and GI. Inter-examiner agreement scores were obtained with the help of a supervisor. For intra-examiner calibration, the researcher took two measurements of five participants’ periodontal parameters, separated by two hours.^32^ Adequate agreement was defined as a kappa value > 0.75 and an interclass coefficient of >90% for clinical parameters. This procedure was carried out to standardize and align the data.

### Real-time PCR

Real-time PCR was employed to detect the presence of P. gingivalis DNA in plaque samples according to the protocol of ABIOpure Total DNA Extraction (ABIOpure; Bothel, WA, USA) and primer preparation, which were supplied in lyophilized form by Macrogen (Macrogen; Seoul, South Korea) (Table 1). The lyophilized primers were dissolved in nuclease-free waterx (NFW) to obtain a final concentration of 100 pmol/μl as a stock solution. A working primer solution of 10 pmol/μl of these primers was prepared by adding 10 μl of primer stock solution (stored at -20°C) to 90 μl of NFW.

**Table 1:** Primers of P. gingivalis

Primer name	Sequence `5-3`	Annealing temp. (°C)
P. gingivalis-F	TGCAACTTGCCTTACAGAGGG	50
P. gingivalis-R	ACTCGTATCGCCCGTTATTC
P. gingivalis-P	Fam-AGCTGTAAGATAGGCATGCGTCCCATTAGCTA
F: forward primer; R: reverse primer.

### Absolute Quantification by the Standard Curve (SC) 

The SC method employs a dilution series of known template copy numbers in the qPCR assay. Linear regression of log concentration (copy µl^-1^) vs CT yields the SC, which is then used to calculate the template concentration (copy µl^-1^) of the sample. For P. gingivalis, nine 0.2 ml tubes were prepared, 90 µl of NFW was added to each tube, 10 µl was added from a sample of 56 x 10^9^ copies µl^-1^ to the first tube, and a serial dilution was made by transferring 10 µl from the first tube to the 2nd tube and so on. The SC reaction started from a tube containing 56 x 10^9^ copies µl^-1^ and yielded 56 copies µl^-1^ in the final tube.

### Statistical Analysis

Data description, analysis, and presentation were performed using SPSS version 22 (Chicago, IL, USA), with presentation as tables and bar graphs. The level of significance was set at p<0.05.

#### Inferential Statistics

One-way ANOVA was employed to compare the means of the test, positive control, and negative control groups at baseline and following treatment. It assisted in determining whether there were statistically significant variations in the groups’ periodontal parameters.

Pearson’s chi-squared test was used to compare the distribution of categorical variables, e.g., gender. If ANOVA detected statistically significant differences, Tukey’s HSD post-hoc test was then utilized to pinpoint particular pairwise differences between the three groups. The paired t-test evaluated the efficacy of the intervention in each group by comparing the means of a single group before and after treatment. The Kruskal-Wallis test was employed to compare differences among the three independent groups. Wilcoxon sum-rank and Wilcoxon signed-rank tests were used for two-group comparison. Pearson’s correlation assess linear relationships between continuous variables.

## RESULTS

### Study Population

The participants were randomly selected 4^th^- and 5^th^-year undergraduate dental students at Mustansiriyah University’s College of Dentistry. The average age of students ranged from 20 to 23 years. Ten men participated in the RSV group, 12 in the positive control (CHX) group, and 11 in the negative control (DW) group according to gender distribution. Six women participated in the placebo group, five in the CHX group, and seven in the RSV group. There was no statistically significant difference in the gender distribution between the groups (p = 0.77; Table 2).

**Table 2:** Demographic data of the participants at baseline

N.	M	F	Pearson’s chi-squared	p-value
%	N.	%	
Groups	RSV	10	58.82	7	41.18	0.51	0.77 NS
CHX	12	70.59	5	29.41
DW	11	64.71	6	35.29
Total	33	64.71	18	35.29
CHX: chlorhexidine; DW: distilled water; F: female; M: Male; NS: non-significant (p>0.05); RSV: resveratrol.

### Clinical Periodontal Parameters

The main result of the study was a decrease in clinical periodontal markers (BOP, PI, and GI) measured at the 1st (baseline) appointment and again 7 days later, after treatment. Clinical periodontal indicators in the three groups did not differ statistically significantly at baseline (Table 3). The 2nd appointment’s findings revealed noteworthy variations in PI, BOP, and GI among the groups. Subsequently, the mean differences between the 1st and 2nd appointments was evaluated, and all the metrics showed statistically significant differences.

**Table 3:** Descriptive and comparative statistics of clinical parameters at the 1st and 2nd appointments

Periodontal parameters	Appointments	RSV	CHX	DW	Tukey’s HSD, statistical significance
PI	PI 1st appointment (mean ± SD)	1.98 ± 0.62	1.86 ± 0.43	2.28 ± 0.35	
PI 2nd appointment (mean ± SD)	1.43 ± 0.58	1.32 ± 0.36	2.20 ± 0.35
PI 1st appointment vs PI 2nd appointment (p-value)	0.00 S	0.00 S	0.00 S	RSV-CHX = 0.77 NS RSV-DW = 0.00 S CHX-DW = 0.00 S
PI groups’ mean difference (mean ± SD)	0.55 ± 0.04	0.54 ± 0.07	0.08 ± 0.00	
Effect size	1.59	1.42	0.81
BOP	BOP% 1st appointment (mean ± SD)	65.87 ± 6.09	57.46± 6.56	76.29 ± 3.68	
BOP% 2nd appointment (mean ± SD)	41.75 ±6.44	32.53 ±4.38	76.05 ± 3.62
BOP 1st appointment vs BOP2nd appointment (p-value)	0.00 S	0.00 S	0.72 NS	RSV-CHX = 0.40 NS RSV-DW = 0.00 S CHX-DW = 0.00 S
BOP% groups’ mean difference (mean ± SD)	24.12 ± 0.34	24.93± 2.18	0.23 ± 0.06	
Effect size	1.55	1.11	0.088
GI	GI 1st appointment (mean ± SD)	1.85 ± 0.50	1.80 ± 0.56	2.16 ± 0.40	
GI 2nd appointment (mean SD)	1.36 ± 0.56	1.23 ± 0.50	2.16 ± 0.29
GI 1st appointment vs GI 2nd appointment (p-value)	0.00 S	0.00 S	0.93 NS	RSV-CHX = 0.71 NS RSV-DW = 0.00 S CHX-DW = 0.00 S
GI groups’ mean difference (mean ±SD)	0.50 ± 0.06	0.57 ± 0.06	0.00 ± 0.11	
Effect size	1.76	1.17	0.02
BOP: bleeding on probing; CHX: chlorhexidine; PI: plaque index; GI: gingival index; RSV: resveratrol; SD: standard deviation; S: signiﬁcant at p<0.05; NS: non-significant at p<0.05.

**Fig 3:**
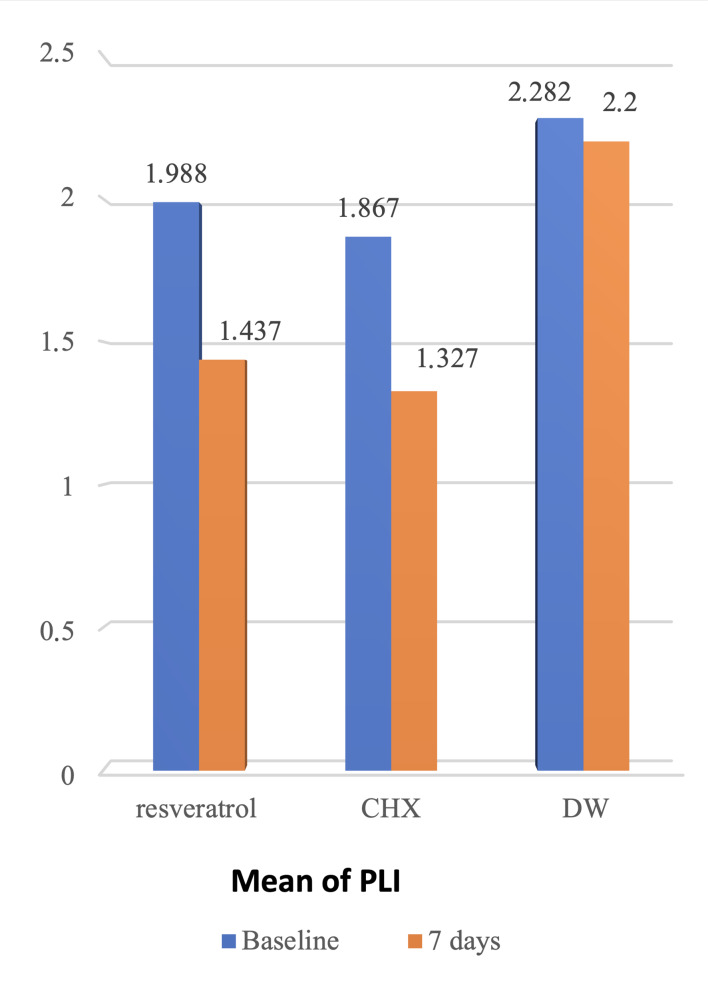

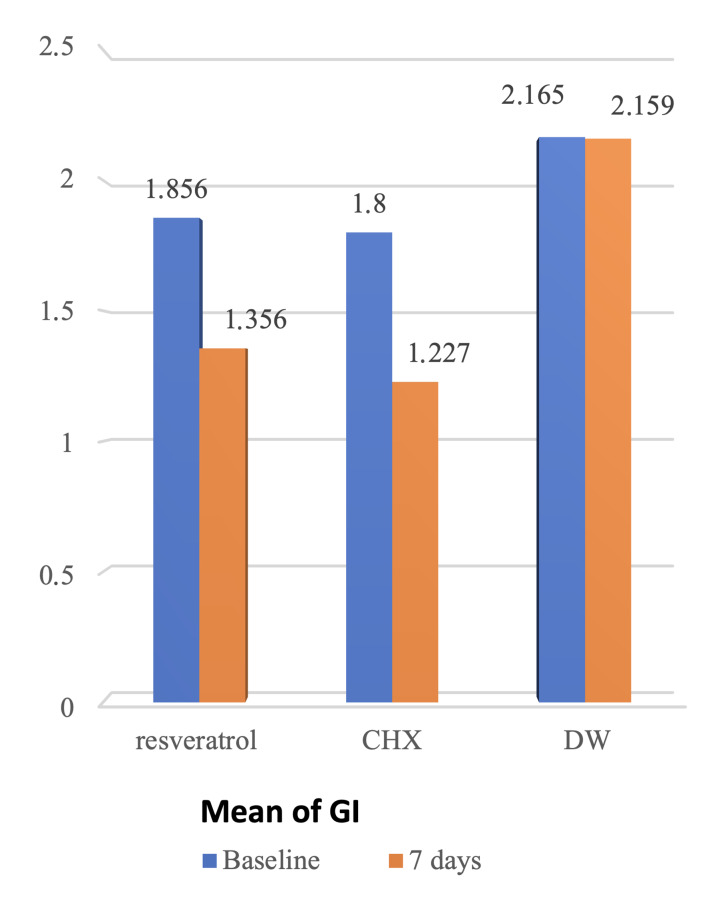

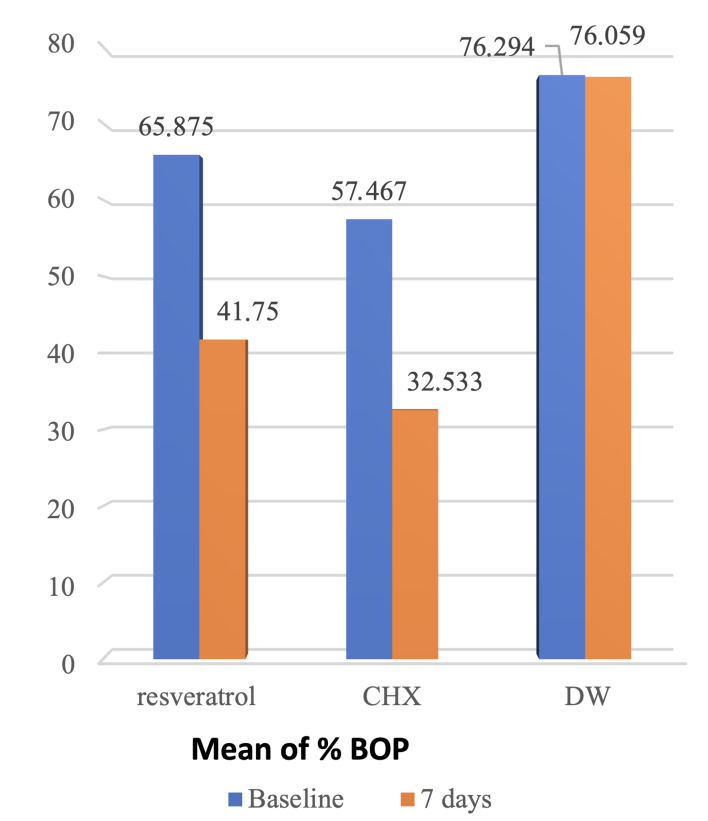
Comparisons of the reduction in the mean of PLI (A), GI (B), and BOP (C) among three groups show a statistically nonsigniﬁcant difference between the RSV group and the CHX group, but a statistically signiﬁcant difference between CHX vs placebo groups and RSV group vs placebo for all parameters.

The RSV and CHX groups did not vary statistically significantly in any of the three periodontal parameters when the two groups were compared independently (p-values for PI = 0.77, BOP = 0.40, GI = 0.72). All periodontal metrics, however, showed statistically significant differences when comparing the RSV group to the placebo group (p-values for PI=0.000, BOP=0.000, and GI=0.000). Additionally, there were notable differences between the CHX and DW groups in all clinical parameters (p-values for PI 0.000, p-value for BOP=0.000) (Fig 3).

### Halitosis Scores 

In addition to improved clinical parameters (secondary outcome), the study’s primary outcome was the reduction in halitosis (organoleptic tongue, organoleptic interdental, and halimeter scores) assessed at the initial appointment (1st appointment) and 1 week later (2nd appointment). At the initial appointment, there were non-significant differences in halitosis scores among the groups (Table 4). At the 2nd appointment, the results showed a statistically significant difference among the groups for all the scores. Subsequently, the groups’ mean difference for the 1st and 2nd appointments was assessed, and all clinical measures showed statistically significant differences. Subsequent analysis of the two groups independently revealed that the halitosis scores of the RSV and CHX groups did not differ statistically significantly (p-value of tongue odor 0.99, floss odor 0.83, and halimeter 0.09).

**Table 4:** Descriptive and comparative statistics of halitosis scores in 1st and 2nd appointments

Halitosis scores	Appointments	RSV	CHX	Placebo	Tukey’s HSD, statistical significance
Organoleptic-tongue	1st appointment (mean ± SD)	3.18 ± 1.10	3.28 ± 0.91	3.11 ± 0.78	
2nd appointment (mean ± SD)	1.81 ± 1.04	1.85 ± 0.77	2.94 ± 0.82
1st appointment vs 2nd appointment (p-value)	0.00 S	0.00 S	0.08 NS	RSV-CHX = 0.99 NS RSV-DW = 0.00 S CHX-DW = 0.00 S
Groups’ mean difference (mean ± SD)	1.37 ± 0.05	1.42 ± 0.14	0.17 ± 2.11	
Effect size	2.22	1.41	0.45
Organoleptic-dental floss odor	1st appointment (mean ± SD)	2.93 ± 0.88	2.64 ± 0.74	2.82 ± 0.52	
2nd appointment (mean ± SD)	1.37 ± 0.77	1.214 ± 0.70	2.76 ± 0.56
1st appointment vs 2nd appointment (p-value)	0.00 S	0.00 S	0.33 NS	RSV-CHX = 0.83 NS RSV-DW = 0.00 S CHX-DW = 0.00 S
Groups’ mean difference (mean ±SD)	1.56 ± 0.11	1.42 ±0.04	0.05 ± 0.03	
Effect size	2.48	1.89	0.24
Halimeter	1st appointment (mean ± SD)	2.64 ± 0.92	2.56 ± 0.81	2.70 ± 0.47	
2nd appointment (mean ±SD)	0.92 ± 0.82	1.50 ± 0.81	2.52 ± 0.51
1st appointment vs 2nd appointment (p-value)	0.00 S	0.00 S	0.18 NS	RSV-CHX = 0.09 NS RSV-DW = 0.00 S CHX-DW = 0.00 S
Groups’ mean difference (mean± SD)	1.71 ± 0.1	1.06 ± 0.00	0.17 ± 0.04	
Effect size	2.80	1.24	0.33
CHX: chlorhexidine; DW: distilled water; RSV: resveratrol; S: statistically signiﬁcant at p<0.05; NS: statistically non-significant at p<0.05.

Nevertheless, all halitosis scores were statistically significantly different between the RSV and DW groups (p = 0.002 for tongue odor, 0.000 for dental floss odor, and 0.001 for halimeters).

**Fig 4:**
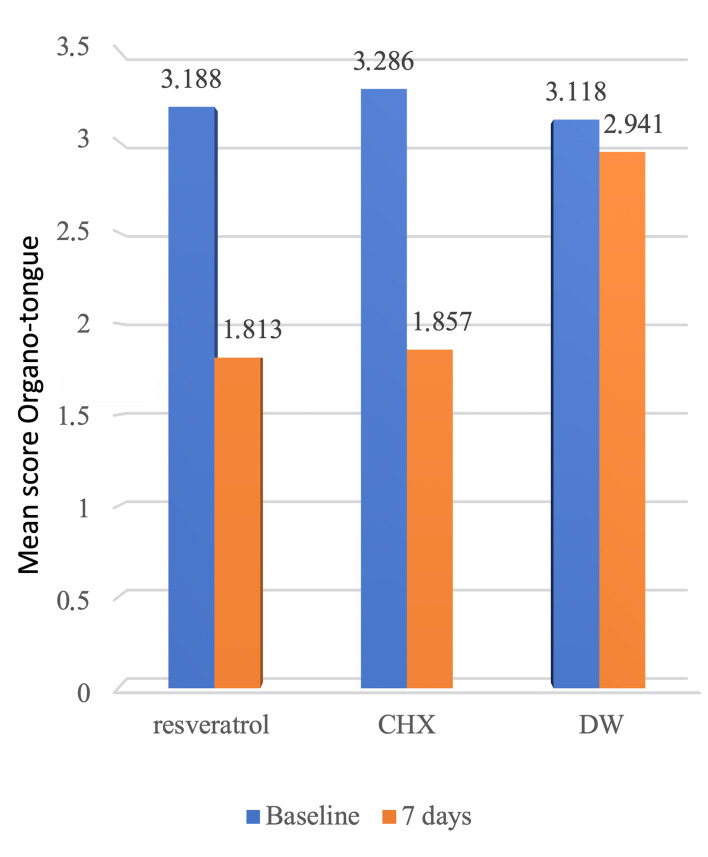

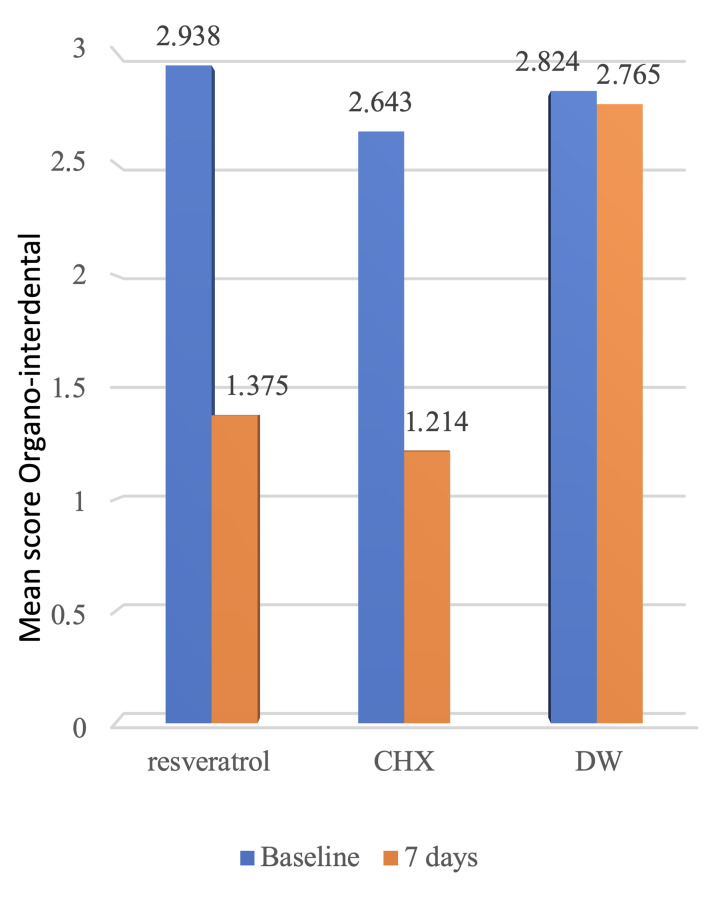

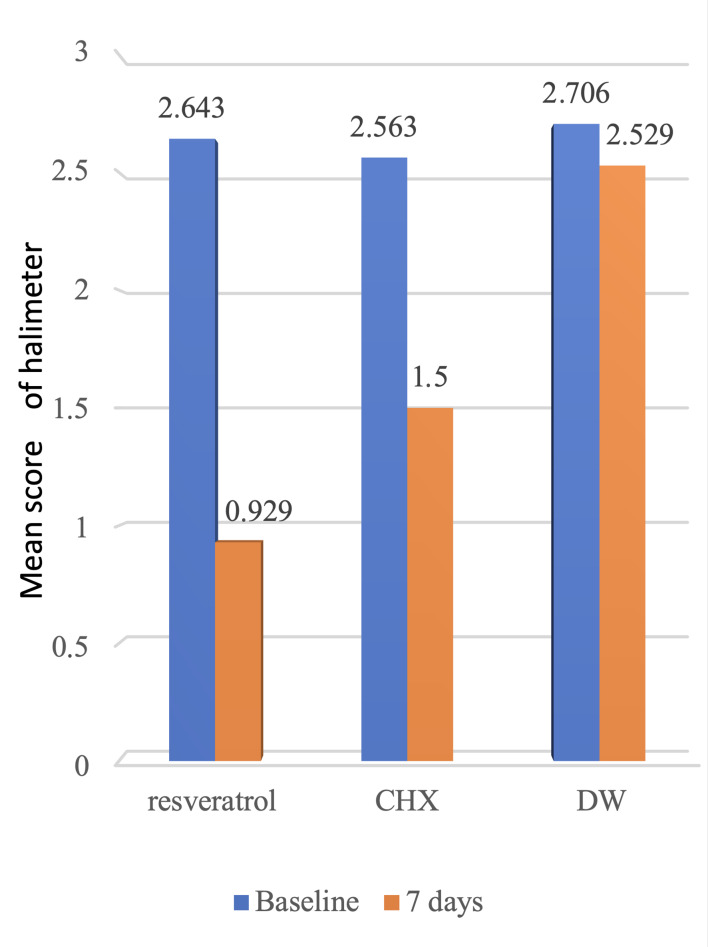
Comparisons of the reduction in the mean of halitosis scores (A), organoleptic tongue odor (B), dental floss odor and (C) halimeter between three groups show a statistically nonsigniﬁcant difference between the RSV and CHX groups, but a statistically signiﬁcant difference between CHX vs placebo and RSV group vs placebo for all parameters.

Statistically significant differences were also found between the CHX and DW groups for all halitosis scores (p-value of tongue odor test 0.004, dental floss odor test 0.000, and halimeter 0.000) (Fig 4).

### Real-time PCR Analysis

**Fig 5:**
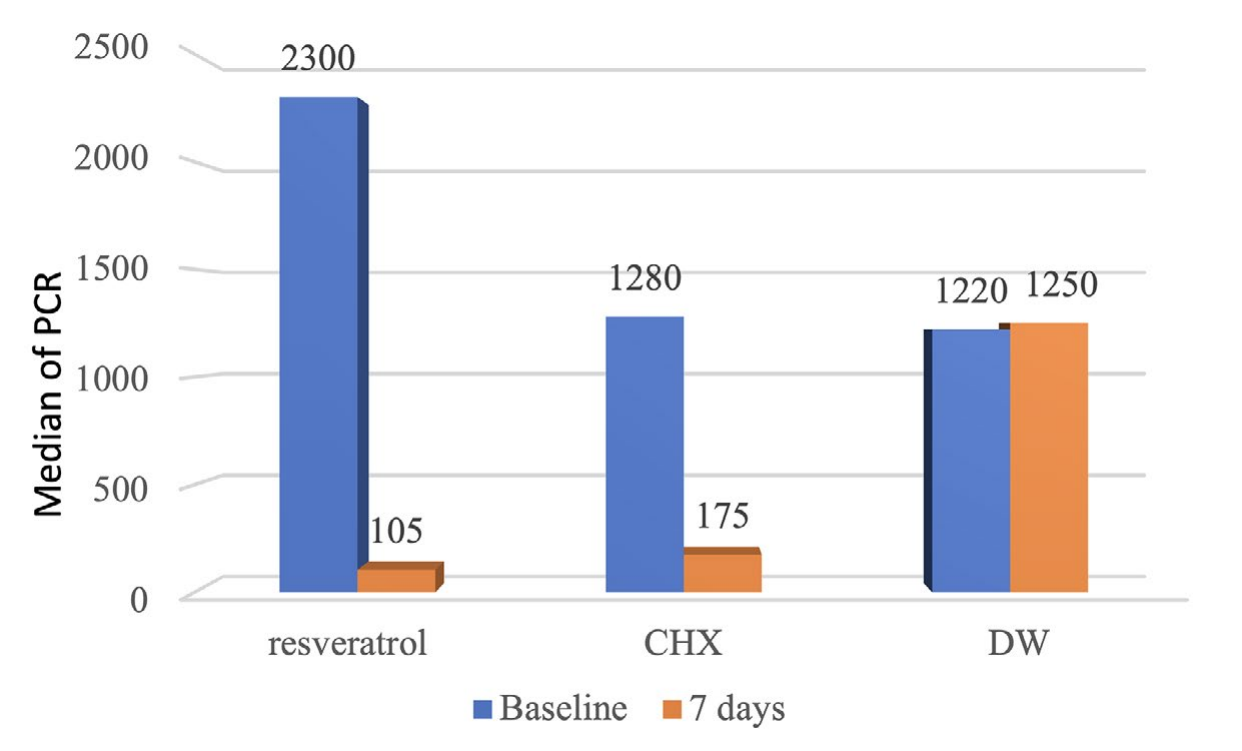
Comparisons of the reduction in the microbial concentration between the three groups show a statistically nonsigniﬁcant difference between the RSV and CHX groups, while showing a statistically signiﬁcant difference between CHX vs placebo and RSV group vs placebo.

The descriptive and inferential statistics of the PCR results for the three groups (RSV, CHX, and DW) at baseline and after seven days of treatment are shown in Table 5 and Fig 5. The numbers obtained from real-time PCR represent the copies of the bacterial gene detected in the sample, reflecting the bacterial load of P. gingivalis in each group. At baseline, all three groups showed high and comparable bacterial counts, with no statistically significant differences among them (Kruskal–Wallis test, p = 0.59). This confirmed that the groups were homogenous in terms of P. gingivalis levels before the treatment. The mean bacterial loads at baseline were as follows: RSV, 1563.26; CHX, 1315.47; DW: 1603.82.

**Table 5:** Descriptive and statistical tests of PCR results among groups and time

	Groups	Kruskal-Wallis	p-value	Wilcoxon sum-rank test
RSV	CHX	DW
Baseline	Minimum	22.49	18.00	125.00	1.04	0.59 NS	
Maximum	3320.00	3265.00	6658.00
Mean	1563.25	1315.47	1603.82
±SD	287.64	287.02	431.71
Median	2300.00	1280.00	1220.00
7 days	Minimum	9.000	5.00	122.00	26.11	0.00 S	RSV-CHX = 0.23 NS RSV-DW = 0.00 S CHX-DW = 0.00 S
Maximum	210.00	2256.00	6600.00
Mean	90.35	382.70	1703.70
±SD	13.77	141.27	426.34
Median	105.00	175.00	1250.00
Wilcoxon signed-rank	3.62	3.24	0.62			
p-value	0.00 S	0.00 S	0.53
Effect size	0.62	0.56	0.11
CHX: chlorhexidine; RSV: resveratrol; DW: distilled water; S: statistically significant at p<0.05; NS: statistically non-significant at p<0.05.

After seven days, a marked reduction in bacterial load was observed in both the RSV and CHX groups, whereas the DW group showed minimal to no improvement. Mean bacterial loads after seven days were: RSV, 90.35; CHX, 382.71; DW: 1703.71. The Kruskal–Wallis test revealed a highly statistically significant difference among the three groups post-treatment (p = 0.000).

Pairwise comparisons indicated that there was no statistically significant difference between the RSV and CHX groups (p = 0.23, NS), suggesting similar efficacy. A highly statistically significant difference was found between the RSV and DW groups (p = 0.000) and between the CHX and DW groups (p = 0.003), confirming the lack of efficacy of DW compared with active treatments. As shown by the Wilcoxon signed-rank test for within-group comparisons, both RSV and CHX groups showed statistically significant reductions in bacterial loads after treatment (p = 0.000 for both). No statistically significant changes were observed in the DW group (p = 0.53). In terms of effect sizes, RSV had a large effect (0.62), CHX a moderate-to-large effect (0.56), and DW a negligible effect (0.11).

### Visual Analog Scale (VAS) Questionnaire

**Fig 6:**
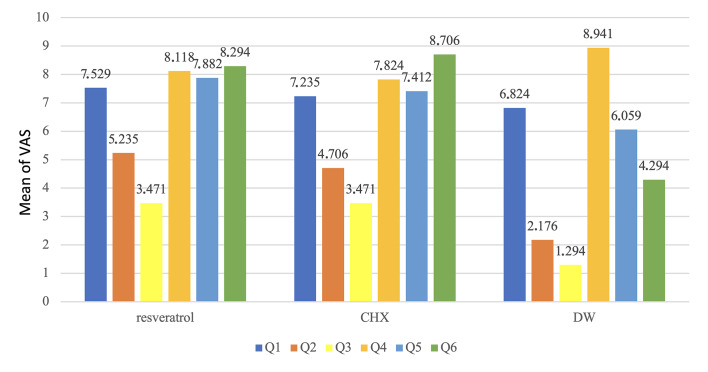
Answers to VAS questionnaire.

Instead of displaying a normal distribution, as indicated in Table 6 and Fig 6, the repeated-measures ANOVA normality test displayed the individuals’ questionnaire answers. Students evaluated the RSV mouthwash (VAS mean 7.53) as not substantially superior to the CHX (VAS mean 7.24) and placebo (VAS mean 6.82) mouthwashes in terms of their answers to the product flavor (Question 1). As stated in Question 2, the duration of taste persistence following usage of RSV (VAS mean 5.24) was statistically significantly longer than with DW (VAS mean 2.18; p 0.002), but not substantially greater than CHX (VAS mean 4.71). Question 3, regarding the impact of mouthwash on food and drink flavors, revealed no discernible differences among RSV (VAS mean 3.47), CHX (VAS mean 3.47), and placebo. Question 4 asked the participants if they found the mouthwashes to cause discomfort. All groups reported a generally high level of comfort, except for some participants in the CHX and RSV groups who experienced itching in the oral mucosa. The results showed that the RSV mouthwash (VAS mean 8.12) did not differ statistically significantly from CHX (VAS mean 7.82) or placebo (VAS mean 8.94). There were no statistically significant differences between RSV, CHX, and placebo in response to mouthwash rinse duration (Question 5). When evaluating mouthwashes’ ability to decrease dental plaque (Question 6), RSV and CHX showed non-significant differences (RSV VAS mean 8.29, CHX VAS mean 8.71), but the placebo was statistically significantly less effective.

**Table 6:** Descriptive and statistical tests of VAS among groups

Groups	Q1	Q2	Q3	Q4	Q5	Q6
RSV	Minimum	3.00	2.00	0.00	0.00	1.00	5.00
Maximum	10.00	10.00	9.00	10.00	10.00	10.00
Mean	7.52	5.23	3.47	8.11	7.88	8.29
±SD	2.15	2.30	3.22	2.95	2.71	1.72
CHX	Minimum	5.00	1.00	0.000	2.00	2.00	5.00
Maximum	10.00	8.00	10.00	10.00	10.00	10.00
Mean	7.23	4.70	3.47	7.82	7.41	8.70
±SD	1.98	2.80	3.65	3.14	2.03	1.49
DW	Minimum	.000	0.000	0.000	8.00	1.00	1.00
Maximum	10.00	9.00	7.00	10.00	10.00	10.00
Mean	6.82	2.17	1.29	8.94	6.05	4.29
±SD	3.12	2.43	1.82	.827	3.09	2.64
F	0.34	7.14	2.96	0.88	2.17	24.90
p-value	0.70 NS	0.00 S	0.06 NS	0.41 NS	0.12 NS	0.00 S
Q: Question; CHX: chlorhexidine; DW: distilled water; RSV: resveratrol; S: statistically significant at p<0.05; NS: statistically non-significant at p<0.05.

## DISCUSSION

Little research has been done on effect of RSV mouthwash on halitosis; this is one of the first studies to do so. One week after baseline, the primary findings of this study showed that the RSV mouthwash improved clinical parameters (PI, BOP, and GI) and decreased the amount of P. gingivalis in dental plaque samples. P. gingivalis was chosen as the target microbe due to its known status as a major pathogen linked to periodontal disease and bad breath. This bacterium allowed a focused assessment of the mouthwash’s antimicrobial activity.

To the best of our knowledge, no prior comparison has been made between RSV and CHX mouthwash for the treatment of halitosis. Therefore, it was not possible to confirm the results directly. The results of the CHX and placebo groups in this study are consistent with earlier research on the adjuvant use of CHX, which demonstrated statistically significant improvements in halitosis, clinical parameters, and microbiological parameters.

There was a statistically significant decrease in the PI readings in the RSV and CHX groups. These results imply that the antiplaque effects of RSV and CHX were comparable. These findings are consistent with a number of studies^7,25^ that suggested RSV might have a limiting effect on cell adhesion molecules; it stops endothelial dysfunction caused by the LPS of P. gingivalis,^13^ which led to notable reductions in gingival inflammation and dental plaque levels when an RSV mouthwash was used. According to the study by Shoukheba and Soheir,^28^ RSV statistically significantly lowered BOP. The improvement in BOP may be due to RSV’s anti-inflammatory effect, which reduces the release of proinflammatory cytokines (IL-1, IL-6, and TNF-α) implicated in the pathophysiology of periodontal disorders.^4,11,17^

The present study demonstrates a greater decrease of P. gingivalis in the RSV group, despite the fact that the difference was not statistically significant, which might be due to its anti-biofilm, antioxidant, and anti-inflammatory qualities, which surpass CHX’s antiseptic action. The effect of RSV on the expression of pathogenic bacteria has been studied for its applications in medical microbiology.^6^ However, previous studies have shown that RSV inhibits cysteine and fimbriae expression in P. gingivalis.^12^ This suggests that resveratrol can prevent bacterial colonization, thereby demonstrating its suitability for treating periodontal diseases. RSV can provide health benefits to patients with periodontal disease by reducing bacterial colonization and biofilm formation via multiple pathways.^35^ Both bacteriostatic and bactericidal activities have been demonstrated previously. This compound has been shown to inhibit major P. gingivalis virulence factors, such as fimbriae and gingipains.^38^ The beneficial properties of RSV identified in the present study will enable future clinical trials on the potential of these bioactive molecules to prevent and/or treat periodontal diseases. It would also be interesting to conduct studies on the advantages provided by the use of oral hygiene products (mouthrinse and chewing gum) or slow-periodontal-release devices (inserted in diseased periodontal sites) containing RSV.

Concerning the students’ feedback about using mouthwash, the 1st question asked about their perception of the mouthwashes’ taste. It is commonly recognized that CHX produces a bitter taste,^2^ which may be caused by CHX interfering with particular taste buds through epithelial ions. Additionally, earlier research^37^ discovered that RSV is linked to bitter-tasting polyphenols. RSV may activate the human bitter taste receptors, TAS2R14 and TAS2R3927. Following product use, the duration of after-taste revealed that RSV and CHX remained tasteable longer than DW did. The participants’ responses to the amount of time spent using CHX were in line with the results of other research.^1^

Regarding the third question, which assessed the participants’ perception of how the mouthwash affected the taste of beverages and food, both the RSV and CHX groups reported moderate interference, whereas the DW group showed a minimal impact. This finding aligns with those of previous studies, in which CHX was often associated with altered taste perception.^34^ Interestingly, RSV showed a similar trend, suggesting that, despite its natural origin, it could have some sensory effects. However, the relatively low mean values across all groups suggest that any perceived impact was mild to moderate. Clinically, this indicates that while taste alteration might be noticed by some mouthwash users, it is unlikely to be severe enough to affect compliance. Further studies with larger sample sizes may provide more definitive evidence of this sensory aspect.

The fourth question evaluated the comfort level perceived by participants during mouthwash use. All groups reported a generally high level of comfort, except for some participants in the CHX and RSV groups who experienced itching in the oral mucosa. These findings indicate that the use of RSV mouthwash was well tolerated and comparable to CHX, which supports its potential as a user-friendly alternative to CHX. Participants perceived that both RSV and CHX mouthwashes effectively reduced dental plaque.

### Limitations

This study has some limitations: First, data from CHX vs placebo were used to determine the sample size. Since RSV is a novel treatment for halitosis, a formal power estimate for this group could not be completed. The final sample size of 54 individuals was sufficient to identify differences between CHX and placebo, but it is still somewhat small, which might make it more difficult to identify RSV’s subtle effects. These results should therefore be regarded cautiously, and larger cohort studies are necessary in the future to assess the effectiveness of RSV in light of the sample’s characteristics.

The research population’s homogeneity is another limitation. The results may not be as applicable to other age groups or populations with varied health statuses, because all participants were young, generally healthy dental students. Future studies incorporating a greater variety of participants are needed to determine whether the reported effects of RSV and CHX on halitosis can be extrapolated to the broader community.

Moreover, the research was based on dental plaque samples, which are often difficult to collect and manage. The process of plaque collection is technically sensitive because of the sticky and minimal nature of dental plaque, and collecting, transferring, and storing the samples without compromising their integrity can be challenging. Additionally, the amount of plaque obtained from each participant was generally small and sometimes uneven, leading to standardization problems, particularly when preparing samples for PCR analysis. Bacterial detection using PCR typically requires a relatively large and consistent amount of plaque. These factors collectively pose limitations in terms of sample consistency and laboratory processing, which may have affected the accuracy and reproducibility of molecular detection outcomes.

A further limitation of this study is the short duration of the intervention, which lasted only 7 days. RSV has antibacterial and anti-inflammatory qualities, but its long-term efficacy and safety as a daily mouthwash substitute have not yet been thoroughly proven; the majority of the evidence is in-vitro or short-term. An extended follow-up period of two to four weeks might yield more therapeutically relevant evidence.

One of the additional active ingredients in the RSV mouthwash (Oroxil, nanotechnology-enhanced) is thyme oil. Its probable antibacterial or odor-reducing properties may have contributed to the observed results, which could be a confounding factor. As a result, it is challenging to assign the benefits exclusively to RSV. To elucidate its independent efficacy, future research employing formulations containing RSV as the single active ingredient is advised. Finally, RSV’s practical usage as a daily oral rinse may be limited by its limited availability and generally higher cost as compared to CHX. However, RSV mouthwash may be a safe and efficient substitute for chlorhexidine in the long-term treatment of halitosis.

## CONCLUSION

Mouthwash containing RSV has a statistically significant effect on P. gingivalis-related halitosis when used as an adjunct to routine oral care. RSV caused a statistically significant decrease in clinical periodontal parameters, including PI, BOP, GI, and halitosis scores, with a noticeable reduction in the level of P. gingivalis. Thus, RSV shows promising short-term efficacy and warrants further longer-term and larger-scale studies.

## ACKNOWLEDGMENT

The authors thank Mustansiriyah University (http://www.uomustansiriyah.edu.iq), Baghdad, Iraq, for their moral support in this study.
